# Working With Type 1 Diabetes: Investigating the Associations Between Diabetes-Related Distress, Burnout, and Job Satisfaction

**DOI:** 10.3389/fpsyg.2021.697833

**Published:** 2021-11-04

**Authors:** Alexandra (Sasha) Cook, Alexander Zill

**Affiliations:** ^1^Psychology Research Institute, University of Amsterdam, Amsterdam, Netherlands; ^2^Department of Experimental and Applied Psychology, Vrije Universiteit Amsterdam, Amsterdam, Netherlands; ^3^Department of Psychology, Technische Universität Chemnitz, Chemnitz, Germany

**Keywords:** diabetes mellitus, burnout, job satisfaction, distress, chronic illness

## Abstract

The present study investigates the association between diabetes-related distress (DD) and work outcomes (burnout and job satisfaction) among employed people with type 1 diabetes. Employed adults with type 1 diabetes (*N* = 297) completed an online survey. Measures assessed emotional, social, food- and treatment-related DD, burnout, and job satisfaction, as well as the type of insulin treatment. We conducted multiple regression analyses to test our hypotheses. Emotional DD was significantly and positively associated with burnout. Social DD was significantly and negatively associated with job satisfaction. The type of treatment (insulin pen versus insulin pump) had no significant effect on the outcomes. This study sets the stage for research on the interactions between working conditions, work outcomes and illness symptoms, and problems of people with type 1 diabetes, and, generally, employees with chronic illnesses. The findings have implications for individual health and illness management, burnout prevention, and occupational health measures.

## Introduction

Diabetes can negatively affect work-life, decreasing the probability of employment and increasing the likelihood of work limitations (Tunceli et al., 2005). Although guidelines summarizing the safety risks of employees with diabetes exist (American Diabetes Association [ADA], 2014), these are limited to the physical risks of diabetes during work (e.g., due to hypoglycemia). Many people with chronic illness continue working after being diagnosed; however, they often experience limitations in their work-life (Vooijs et al., 2015). Life with a chronic health condition is not only characterized by physical symptoms and impairments but requires specific illness or health management (Rak, 2014). Illness or health management refers to all activities to maintain or improve one’s health and prevent adverse health consequences, such as following a medical treatment plan and attending appointments with medical professionals (McGonagle et al., 2020).

For employed people with type 1 diabetes, health management comprises controlling blood sugar levels and the intake of insulin (Rak, 2014). However, the requirements and demands caused by health and illness management can present a challenge to employed people with diabetes, as they need to balance both the demands of their job as well as those of the illness when allocating their time and energy resources (McGonagle et al., 2020), which poses a risk of negative work-related outcomes, in particular burnout (Demerouti et al., 2001).

Burnout is associated with negative occupational consequences, such as lower retention rates (Rabatin et al., 2016), lower job performance (Taris, 2006), and withdrawal (Taris et al., 2001), and psychological consequences, such as lower life satisfaction and depressive symptoms (Hakanen and Schaufeli, 2012). Furthermore, burnout can have physical consequences, particularly affecting the metabolic and cardiovascular systems (Kitaoka-Higashiguchi et al., 2009), leading to substantial health risks for people with type 1 diabetes.

Due to the early onset age and the necessity of insulin treatment (Maahs et al., 2010), most employees with type 1 diabetes may face the challenge of integrating both work and illness management for most of their time as active members of the workforce. Thus, insights into the association between diabetes type 1 and burnout are crucial for developing appropriate working conditions for chronically ill people because certain working conditions (e.g., lack of autonomy in assembly-line work or customer service) make the illness management of employees with type 1 diabetes more difficult. Moreover, various preventative countermeasures in health education can be derived for different stakeholders, e.g., implementing educational training for leaders and HR managers and specific stress prevention training for employees with diabetes. However, even though the interest in mental health at work is steadily rising, to this point, there is only little insight into the association between chronic illness and work outcomes such as burnout and job satisfaction. This is problematic as people with chronic illnesses such as diabetes are often more likely to retire earlier, leading to an economic and societal burden (Vijan et al., 2004), and both burnout and job satisfaction are important predictors of workplace retention (Rumrill et al., 2004; Swider and Zimmerman, 2010). Furthermore, existing studies on work and diabetes have focused mostly on diabetes mellitus as a potential health outcome of work stress (Cosgrove et al., 2012) or shift work (Gan et al., 2015).

The present paper investigates the association of diabetes-related distress (DD) on burnout and job satisfaction at work compared to other work-related demands (e.g., quantitative job demands and lack of autonomy). Applying resource-based models of occupational health and burnout, namely the Job Demands-Resources Model (JDR, Bakker et al., 2014) and the Conservation of Resources Model (COR, Hobfoll and Freedy, 1997), we investigate the association between perceived illness-related distress and work-related mental health outcomes. In line with recent additions to the Job-Demands Resources model (Demerouti et al., 2001) that emphasize the role of personal resources (Schaufeli and Taris, 2014), we add to the literature by investigating the employees’ existing health, respectively illness status as a personal resource (McGonagle et al., 2015). We tested our assumptions utilizing a cross-sectional online study amongst employed people with type 1 diabetes. Our findings offer a potential starting point to better understand employees with type 1 diabetes at work. Moreover, they could give a push to engage and research regarding counseling, medical treatment, the improvement of existing workplace health management programs, the development of new workplace health management programs aimed at providing support for chronically ill employees, and human resource development measures for supervisors and managers of a health-diverse workforce. Furthermore, our study responds to the call for more theory-driven research regarding the work-life and careers of people with chronic diseases (Lehmann et al., 2021).

## Diabetes-Related Distress

An important factor to consider when studying the effects of a chronic illness is that people can strongly differ regarding their individual and subjective problems with the respective disease and may experience different effects of these problems. The most prominent variable to capture the inter-individual variance regarding these experiences is assessing the level of individually perceived DD. DD comprises the “unique and hidden emotional burden or frustration that comes with living with diabetes and considers ongoing concerns, worries, and fears of diabetes management as well as diabetes complications” (Abdoli et al., 2019, p. 2). Although DD is often utilized and conceptualized as a construct with a single general factor, factor analyses suggest it can be differentiated into four subordinate dimensions: Emotional problems, food-related problems, treatment problems, and social problems (lack of support) (Polonsky et al., 2005). The general measures of DD and the emotional and treatment-related subdimensions of DD are positively associated with glycosylated hemoglobin (HbA_1__c_) levels and blood glucose self-monitoring. The HbA_1__c_ level “reflects average plasma glucose over the previous eight to 12 weeks” (World Health Organization [WHO], 2011, p. 6) and is commonly used as a diagnostic measure for diabetes. However, it is important to note to this point there is no theoretical framework that supports the inclusion and definition of the subdimensions. Furthermore, there is conflicting evidence regarding the factor structure of DD assessments, as some studies (e.g., Graue et al., 2012) have failed to replicate the four-factor solution or found a one-factor general DD solution to have a better fit (Graue et al., 2012; Schmitt et al., 2016).

People with type 1 diabetes report higher general DD compared to people with type 2 diabetes, and the level of perceived diabetes distress is higher amongst people with more diabetic complications such as kidney damage, albuminuria, retinopathy, neuropathy, heart disease, stroke, and vascular disease (Fenwick et al., 2018). People with higher levels of general DD report lower subjective health, as well as more problems with self-care, dieting, and blood glucose testing (Schmitt et al., 2016) as well as higher Hb1Ac (Graue et al., 2012) and more fear of hyperglycemia (Amsberg et al., 2008). General DD is also associated with depressive symptoms, general anxiety, and lower self-esteem (Fenwick et al., 2018) and coping styles such as distractive coping, trivialization, and depressive coping (Schmitt et al., 2016). Individuals experiencing higher levels of DD also tend to experience a higher stigma associated with type 1 diabetes, including the perception that they are being treated differently due to their illness and that they are blamed and judged (Browne et al., 2017).

As studies investigating the effects of the subdimensions are sparse, there is little evidence of differences in their effects. All four facets are negatively correlated with well-being and positively associated with worrying about hyperglycemia and trait anxiety (Snoek et al., 2000). Furthermore, all four subtypes are significantly and positively associated with depressive symptoms (Polonsky et al., 2005; Martin et al., 2018) and negatively associated with mental health, social functioning, and vitality (Graue et al., 2012). However, only emotional and treatment-related DD are significantly associated with HbA1C and self-monitoring of blood glucose (Snoek et al., 2000). Individuals with higher emotional, social, and food-related DD also report significantly greater problems regarding meal planning. Furthermore, whereas food-related and interpersonal/social DD are positively associated with higher cholesterol levels, only food-related DD is associated with lower self-monitoring of blood glucose (Polonsky et al., 2005).

## Associations of Diabetes-Related Distress and Work Outcomes

Research on the work-life of people with type 1 diabetes is sparse, however, is evidence that “working-age adults with diabetes are more likely to be unemployed or unable to work, miss workdays, or have severe difficulty with work tasks compared to those without diabetes” (Fritschi and Quinn, 2010, p. 37). In a qualitative study on diabetes and work, young adult employees with type 1 diabetes reported difficulties with diabetes management during work. Especially under time pressure or when experiencing difficulties with the illness, employees with type 1 diabetes reported that they neglected or decreased the diabetes management activities (Balfe et al., 2014). Previous research also indicates a negative association between the number of years since the onset of the illness and wages, whereas a stable HbA1c was positively associated with the wage level (Brown et al., 2012). Furthermore, a study including people with type 1 and type 2 diabetes found that working conditions characterized by high job demands, low decision latitude, and low social support predicted fatigue amongst the study sample (Weijman et al., 2003). A study on the association between burnout and receiving treatment for chronic illnesses indicates an association between burnout and treatment for diabetes. However, the authors did not indicate the diabetes type (De Beer et al., 2016).

Insights into the effects of diabetes on general well-being and everyday functioning indicate two main mechanisms through which type 1 diabetes may impact employment and lead to negative work-related outcomes. The first mechanism is rooted in the effect of disease on the perceived energy and emotional well-being. Existing evidence implies that type 1 diabetes is linked to chronic fatigue, i.e., feelings of physical and emotional exhaustion (Goedendorp et al., 2014; Kalra and Sahay, 2018). Chronic fatigue in people with type 1 diabetes is only weakly associated with blood glucose levels. It cannot be fully explained by depression (Goedendorp et al., 2014), a common comorbidity of type 1 diabetes (Gendelman et al., 2009). However, fatigue can have substantial effects on daily life, as it is related to reported functional impairments, such as mobility, social interactions, and work limitations (Goedendorp et al., 2014). A theoretical framework on fatigue in people with diabetes (Fritschi and Quinn, 2010) states that DD is one of the psychological factors associated with fatigue. However, it does not state an exact causality relation between the concepts.

The second mechanism is rooted in the burden of treatment, i.e., the time and effort required to manage a chronic illness (Sav et al., 2015). To prevent potentially fatal exacerbations, type 1 diabetes requires a high and constant level of day-to-day management activities, including, but not limited to, blood glucose monitoring. The responsibility of the management activities lies mainly on the people with type 1 diabetes themselves (Ahola and Groop, 2013). As diabetes self-management requires resources such as time, energy, and cognitive capacity, employed people with type 1 diabetes can experience tensions between their work and diabetes management (Pyatak, 2011). Moreover, people with type 1 diabetes may experience a lack of mental, emotional, and physical energy and feelings of detachment regarding their diabetes management, a phenomenon known as diabetes burnout. Diabetes burnout is strongly and positively associated with DD. However, it is conceptually distinct (Abdoli et al., 2020). In sum, both mechanisms imply that employees with type 1 diabetes experience a loss of resources in terms of time and energy due to the symptomology of the illness itself and the high and constant illness management requirements.

Individual resources are highly relevant in theories explaining work-related employee well-being and other, more distal work-related outcomes such as performance and satisfaction. Two fundamental theoretical frameworks from the field of occupational health psychology, namely the conservation of resources theory (COR) (Hobfoll, 1989) and the job-demands resources model (JDR) (Demerouti et al., 2001), include employee resources as an essential factor in their explanations of the etiology and processes underlying employee strain. As the development of the JDR was influenced by COR (Bakker and Demerouti, 2017), both theoretical frameworks lead to similar conclusions regarding their proposed effects of employee resources, or a lack thereof, on strain, which manifests as burnout. Burnout is one of the most commonly researched work-related mental health outcomes and is mainly characterized by exhaustion, loss of energy, depletion, and detachment (Maslach and Leiter, 2017).

The COR focused on the effects of the availability and investment of resources which can include material objects (e.g., money), but also personal characteristics (e.g., self-esteem), and energy resources (Hobfoll, 1989; Park et al., 2014). According to COR, a loss of resources is stressful to individuals and predicts strain and burnout (Hobfoll, 1989). As the resources of employees with type 1 diabetes resources can be limited due to the illness and its symptoms (e.g., fatigue) (Kalra and Sahay, 2018), as well as the time and effort required to manage the illness (burden of treatment) (Sav et al., 2015), they may have a higher risk for a resource loss cycle. Higher levels of DD should intensify this resource loss cycle and hence the level of burnout, particularly because high diabetes distress is associated with negative emotionality and perceived stress (Coccaro et al., 2020).

In contrast to the COR, the JDR model considers the work characteristics as it postulates that stress and burnout are the results of direct effects and interactions of job demands and job resources. High job demands (e.g., time pressure, physical workload, shift work) directly affect employee strain. A lack of job resources (rewards, job control, support) or personal resources (resilience, self-efficacy, and intrinsic motivation) can lead to increased difficulties in meeting the job demands, thus also increasing the risk for burnout (Demerouti et al., 2001; Schaufeli and Taris, 2014).

The JDR model is one of the most frequently applied models to explain the development of stress and burnout (Schaufeli and Taris, 2014). In a recent study amongst employed people with multiple sclerosis (MS), a chronic neurological autoimmune illness, job demands and job resources predicted the experienced MS-related difficulties at work. These difficulties (e.g., cognitive and physical limitations and external barriers) mediated the effects on job demands and burnout and job demands and turnover intentions (Lehmann et al., 2021). Whereas this study investigated the health-related difficulties at work as a mechanism within the JDR model, physical health status can also be conceptualized as a personal resource within the JDR framework. Thus, a pre-existing chronic illness or health impairment signifies a loss of resources or status of diminished resources, leading to a higher vulnerability toward demands and stressors (McGonagle et al., 2015).

Previous research on diabetes (type 1 and type 2) shows inconclusive results on the association between diabetes (versus no diabetes) and burnout. In general, burnout seems to be more prevalent among employees with chronic medical illnesses than employees without any chronic medical condition (Armon et al., 2014). A study including 7895 employees from different sectors (De Beer et al., 2016) did not find a significant relationship between diabetes and levels of burnout. However, the authors did not assess or report the type of diabetes among the participants.

To our knowledge, only one existing study has investigated the effects of the individual perception of the severity of the illness in terms of DD and occupational burnout. In a sample of employees with type 2 diabetes, self-reported DD was not only significantly associated with burnout but also mediated the effect of Hb1ac levels on burnout and the effect of positive affect on burnout. Moreover, it was particularly strongly associated with the exhaustion dimension of burnout (Han, 2008). According to COR and JDR, we assume that high levels of DD are associated with high levels of burnout. Moreover, we suggest that DD explains variance in burnout above and beyond other job characteristics.

*Hypothesis 1: Diabetes-related distress is positively associated with burnout.*

In addition to burnout, we are focusing on job satisfaction as a secondary outcome. Job satisfaction is a positive emotional attitude or state resulting from the appraisal of one’s job or experiences on the job, that is influenced by dispositions of the employee and job characteristics (Judge et al., 2020). Meta-analytical evidence (Faragher et al., 2005) shows strong and negative associations with burnout, positive associations with mental health outcomes (anxiety and depression), and smaller yet significant associations with subjective physical illness. Job satisfaction is also an important predictor of turnover intention and job tenure, but it also plays an important part in work adjustment and rehabilitation for people with chronic diseases (Roessler et al., 2004).

Furthermore, job satisfaction is strongly and positively associated with self-esteem (Faragher et al., 2005). A study on self-esteem and type 1 diabetes showed that individuals that reported feeling overwhelmed by diabetes also had low levels of self-esteem. Moreover, self-esteem and illness self-concept were positively related to diabetes-related problems. Individuals with low levels of self-esteem reported experiencing less support and more treatment- and emotional problems 5 years later (Luyckx et al., 2008). As self-esteem is related to diabetes-related problems and job satisfaction, we assume that DD affects job satisfaction negatively and explains variance in job satisfaction above and beyond other job characteristics.

*Hypothesis 2: Diabetes-related problems are negatively associated with job satisfaction.*

Regarding the dimensionality of DD, there are no clear theoretical and empirical indicators that allow for the development of distinct hypotheses for the specific subdimensions, particularly as all four subdimensions are positively associated with mental health problems and negatively associated with social functioning (Graue et al., 2012). Given the limited evidence so far, we, therefore, aim at an explorative analysis of the associations between different types of DD and work-related outcomes.

*Research Question 1: Are there differences between the subdimensions regarding their association with burnout?*
*Research Question 2: Are there differences between the subdimensions regarding their association with job satisfaction?*

## Types of Diabetes Treatment

Individuals with type 1 diabetes have two main options for insulin treatment: multiple daily injections of rapid-acting insulin combined with daily basal insulin or continuous subcutaneous insulin infusion (Maahs et al., 2010). Past research investigated the effect of different insulin treatments (syringe, pen, and pump) on psychological outcomes: a study on 132 patients with insulin-dependent diabetes mellitus investigated insulin therapy change. Patients who changed from traditional syringe treatment to insulin pen were more satisfied with their performance at work, life in general, and time for diabetes management and felt less restricted regarding social relationships, diet, leisure time. Patients who changed from an insulin pen to an insulin pump were more satisfied with time for diabetes management and felt less restricted regarding social relationships, diet, leisure time (Chantelau et al., 1997). These findings suggest that insulin treatment with a pump can improve illness management, which should lead to less DD and hence lower levels of burnout and higher levels of job satisfaction.

*Hypotheses 3a: Employees who use an insulin pump in contrast to an insulin pen report lower levels of burnout.*
*Hypotheses 3b: Employees who use an insulin pump in contrast to an insulin pen report higher levels of job satisfaction.*

## Materials and Methods

### Study Design

We conducted a cross-sectional study among adult employed people with type 1 diabetes. Before starting the questionnaire, the participants had to confirm that they fulfilled the inclusion criteria (minimum age of 18 years, employment with at least 20 h per week work time, medical diagnosis of type 1 diabetes). The inclusion criteria were presented to the participants as a list and the participant and to select the option stating that they fulfilled all criteria before they could proceed with the survey.

We conducted an *a priori* power analysis using G^∗^Power (Faul et al., 2007) to determine the sample size. Based on previous similar research on chronic illness severity, burnout, and job satisfaction (Han, 2008; Siu et al., 2013), we chose a size of *f*^2^ = 0.08 as the basis of our calculation resulting in a minimum sample size of *N* = 155 to reach a power of 0.80 and *N* = 238 for a power of 0.95 assuming that we would test the effects of the four DDS subdimensions and their incremental effects above and beyond six covariates. We therefore aimed at acquiring a sample size between 200 and 300.

Data collection took place in the first quarter of 2018 (early January until late March). We recruited the sample through an announcement by a German monthly magazine for people with diabetes and social media groups on diabetes. The assessment was carried out via a self-report online questionnaire and was only available in German. Participants were excluded if they did not have type 1 diabetes, suspected having type 1 diabetes yet did not have a diagnosis from a medical professional, were self-employed or unemployed, and/or did not speak German. Participants did not receive compensation for their participation in the study. The study design was submitted for a pre-review to the responsible ethics review board, which declared no necessity for a full review.^1^ During the design and conduction of the study, we made sure that we adhered to the ethical guidelines of the German Psychological Association (DGPs).

### Measures

#### Burnout

We assessed burnout with the personal burnout scale of the Copenhagen Burnout Inventory (CBI, Kristensen et al., 2005). We used the German translation of the scale as included in the German version of the Copenhagen Psychosocial Questionnaire (Nübling et al., 2006). The scale consists of 6 items answered on a 5-point Likert-type scale from 1 = does not apply at all, to 5 = applies very strongly. We calculated the mean value of the scale. Higher mean values imply more severe burnout symptoms. The scale showed very good reliability with α = 0.92, respectively ω_RT_ = 0.94 (Revelle, 2016; McNeish, 2018).

#### Job Satisfaction

We assessed job satisfaction with the eponymous subscale from the COPSOQ (Nübling et al., 2006). The scale consists of nine items that ask for the participant’s satisfaction with different job-related aspects (e.g., salary, general working conditions, the way his/her abilities are used) with a 4-point Likert-type response scale from 1 = very dissatisfied to 4 very satisfied. Larger scale mean values imply higher job satisfaction. The scale showed good reliability with α = 0.86 and ω_RT_ = 0.90.

#### Diabetes-Related Distress

We assessed DD with the German version of the Problem Areas in Diabetes Questionnaire (PAID, Polonsky et al., 1995). The PAID questionnaire is frequently used amongst individuals with type 1 diabetes to assess DD and has been translated into multiple languages (El Achhab et al., 2008; Lee et al., 2015). The PAID has four subscales that assess the four different areas of diabetes-related problems: emotional (12 items), social (two items), food-related (three items), and therapy-related problems (three items) (Snoek et al., 2000). All items were answered in a 4-point Likert-type scale from 1 = does not apply at all to 5 = applies very strongly. We calculated the mean values for each subscale, with higher values signifying higher levels of DD. The scale reliabilities were acceptable to excellent, with α = 0.93 and ω_RT_ = 0.95 (emotional), α = 0.73 and ω_RT_ = 0.73 (social), α = 0.76 and ω_RT_ = 0.76 (food-related), and α = 0.70 and ω_RT_ = 0.73 (therapy-related).

#### Type of Insulin Therapy

We assessed the type of insulin therapy with a single item, asking the participants to check the type of therapy they currently apply (0 = pen, 1 = pump, 2 = syringe, 3 = other). Participants that chose the “other” category were asked to describe their insulin therapy in an open text field.

#### Control Variables

We included age and gender as covariates as women and younger employees are more likely to experience higher emotional exhaustion (Brewer and Shapard, 2004; Purvanova and Muros, 2010). To analyze the incremental validity of the predictors above and beyond working conditions whether the participant’s job included leadership responsibility. We assessed leadership responsibility with one item: “What is your current job position” with two response options 1 = employee with leadership responsibility and 0 = employee without leadership responsibility. Furthermore, we assessed quantitative job demands and the degree to which the participants had control over their work time using two eponymous scales from the COPSOPQ (Nübling et al., 2006). The quantitative job demands scale consisted of seven items with a 5-point Likert-type response scale from 1 = never to 5 = always. Higher mean values imply higher perceived quantitative job demands. Scale reliability was excellent with α = 0.86 and ω_RT_ = 0.90. The control over work time scale consisted of four items and was answered with the same response scale as the quantitative job demands scale. Scale reliability was good with α = 0.87 and ω_RT_ = 0.90.

### Sample

The final sample consisted of *N* = 237 participants. Of the study sample, 67.34% were female, and 46.80% reported having a university degree. Participants reported working an average of 35.82 h/week (*SD* = 7.05). About half of the participants (*N* = 156, 52.2%) were employed in the public sector, respectively worked in the fields of healthcare, education, whereas *N* = 46 (15.5%) worked in manufacturing, *N* = 29 (9.8%) worked in trade, transport, or the hospitality industry, *N* = 26 (8.8%) worked in the information and communication industry, and *N* = 24 in the financial, insurance and business services.

The rest of the sample consisted of employees from the energy, real estate, and agricultural sectors. Most participants reported having disclosed their diabetes to their line manager (93.94%) and at least some colleagues (95.96%). Fifty-nine participants (19.87%) reported having a secondary illness (38.98% diabetic retinopathy, 30.51% diabetic neuropathy, 3.39% diabetic nephropathy, and 25.42% other/not stated). 46.46% reported using a pen, and 53.53% reported using a pump. None of the participants in this sample reported using a different type of insulin therapy.

### Analytical Approach

The steps of the data analysis were planned as follows: first, a confirmatory factor analysis (CFA) was to be conducted to confirm the validity of using the four DD subscales (compared to a one-factor solution), followed by a descriptive analysis of the bivariate correlations (for the numeric variables) and *t*-tests to analyze possible associations between dichotomous and numerically scaled variables. To test the study hypotheses, we conducted a series of multiple regression analyses for each outcome. In the baseline models, we regressed the respective outcome on age and gender only (Models 1a and 2a). In the next step, we added the work-related covariates (Models 1b and 2b) before adding the main predictors in the final step (Models 1c and 2c). All analyses except for the CFA were carried out using the psych package (Revelle, 2021) for the R environment (R Development Core Team, 2015), whereas the CFA was conducted using the lavaan package for R (Rosseel, 2012).

## Results

Before hypothesis testing, we conducted a confirmatory factor analysis to confirm the 4-factor structure of the PAID. We compared the proposed 4-factor structure to a single-factor model. The 4-factor structure fit the data significantly better, with Δχ^2^_(__6__)_ = 129.21, *p* < 0.001. However, it is important to note that the 4-factor model did not meet the criteria for an acceptable model fit (cf. Schermelleh-Engel et al., 2003), with χ^2^_(__164__)_ = 592.93, CFI = 0.887, and RMSEA = 0.094 (Table 1).

**TABLE 1:** Results of the confirmatory analyses testing the 4-factor structure of the PAID.

	*X* ^2^	*df*	*X*^2^/*df*	CFI	RMSEA	Δ*X*^2^	Δ*df*
1-factor model	722.14	170	4.25	0.855	0.105	129.21***	6
4-factor model	592.93	164	3.62	0.887	0.094		

***p < 0.001.

The analysis of the bivariate correlations (Table 2) revealed that the number of working hours per week was not significantly associated with any of the study outcomes or predictors and was therefore not included in the further analyses. The correlation analyses further revealed positive and significant bivariate associations between all dimensions of DD and burnout and significant negative associations between DD and job satisfaction. Gender was significantly correlated with emotion-related DD and burnout, indicating that female participants reported higher levels of both variables. Control over work time was significantly and negatively correlated to all four DD dimensions, and quantitative work demands were positively correlated with food-related DD. In addition to the bivariate correlations, we conducted t-tests to investigate whether gender was associated with the study variables. Participants identifying as female reported significantly higher levels of burnout (*M* = 3.15) compared to participants identifying as male (*M* = 2.78), with *t*(295) = 3.58, *p* < 0.001. There was no significant difference in job satisfaction between genders, with *t*(295) = −1.05, *p* = 0.30. (See Supplementary Appendix A for the gender differences of all study variables).

**TABLE 2:** Bivariate correlations of the numerically scaled variables.

Variable	*M* (*SD*)	1	2	3	4	5	6	7	8
(1) Age	41.25 (11.09)								
(2) Quantitative job demands	3.10 (0.74)	0.05							
(3) Control over work time	3.42 (1.09)	0.16*	−0.29**						
(4) Emotional DD	2.44 (0.92)	−0.17**	0.08	−0.39**					
(5) Social DD	1.97 (1.03)	−0.17**	0.08	−0.39**	0.70**				
(6) Food-related DD	2.46 (1.01)	−0.13*	0.13*	−0.36**	0.74**	0.50**			
(7) Therapy-related DD	1.91 (0.88)	−0.22**	–0.01	−0.34**	0.67**	0.61**	0.47**		
(8) Burnout	3.03 (0.85)	–0.11	0.20**	−0.40**	0.70**	0.55**	0.52**	0.48**	
(9) Job satisfaction	3.50 (0.73)	0.03	−0.32**	0.44**	−0.42**	−0.42**	−0.37**	−0.33**	−0.40**

N = 297, *p < 0.05, **p < 0.01.

We conducted *t*-tests to test for differences in the outcomes as a function of the type of insulin therapy. There were no differences in either burnout, *t*(295) = −0.81, *p* = 0.42, or job satisfaction, *t*(295) = 0.65, *p* = 0.52, between participants using a pen and participants with a pump (see Supplementary Appendix B for the differences between pen and pump for all study variables).

To test the study hypotheses, we conducted a series of multiple regression analyses for each outcome. In the baseline models, we regressed the respective outcome on age and gender only (Models 1a and 2a). In the next step, we added the work-related covariates (Models 1b and 2b) before adding the main predictors in the final step (Models 1c and 2c). The results of the regression analyses are stated in Table 3.

**TABLE 3:** Models regressing the standardized outcomes on standardized predictors.

	Outcome: burnout			Outcome: job satisfaction		
	Model 1a	Model 1b	Model 1c	Model 2a	Model 2b	Model 2c
Intercept	−0.27**	–0.11	–0.13	0.08	–0.12	–0.10
Sex	0.40**	0.24	0.20*	–0.12	0.07	0.10
Age	–0.05	–0.02	0.06	0.02	–0.02	–0.07
Leadership		–0.24	−0.28**		0.34**	0.35**
Quantitative work demands		0.12*	0.14*		−0.23**	−0.24**
Control over work time		−0.33**	–0.06		0.37**	0.21**
Type of therapy			0.10			–0.08
Emotional DD			0.62**			–0.13
Social DD			0.09			−0.19**
Food-related DD			–0.01			–0.03
Therapy-related DD			–0.01			–0.07
*R* ^2^	0.04	0.18	0.54	0.00	0.24	0.34

N = 297, *p < 0.05, **p < 0.01; gender coded: 0 = male, 1 = female; leadership coded: 0 = no leadership responsibility, 1 = leadership responsibility; type of insulin therapy coded 0 = pen, 1 = pump.

Controlling for demographic variables (age and gender) as well as work-related variables, emotional DD was significantly and positively associated with burnout (β = 0.62, *p* < 0.001), thus supporting Hypothesis 1. Together, all DD variables explained 36% of the variance in burnout. Finally, social DD was significantly and negatively associated with job satisfaction (β = −0.19, *p* < 0.01), controlling for age, gender, and working conditions. Together, all DD variables explained 10% of the variance in job satisfaction. Regarding Hypotheses 3a and 3b on the association between type of insulin therapy and work-related outcomes, we did not find any indication of a difference in burnout or job satisfaction between participants using insulin pens and participants with pumps in the regression analysis. These results align with the results of the *t*-tests that we carried out in the descriptive analyses. Thus, we conclude that Hypotheses 3a and 3b are not supported.

Similar to the results of the bivariate correlation, participant gender had a positive and significant effect on burnout in the regression analyses, implying that female participants were more likely to report higher burnout levels. Quantitative job demands, which were included as a covariate, had a significant positive effect on burnout and a significant negative effect on job satisfaction. In contrast, control over work time was significantly and positively related to job satisfaction only.

### Supplemental Analyses

Due to the cross-sectional nature of the assessment and the strong positive correlation between emotional DD and burnout, we conducted a confirmatory factor analysis to justify treating these variables as separate constructs. We tested a single factor model (emotional DD and burnout) against the 2-factor model (Table 4). The 2-factor model fit the data significantly better, with Δχ^2^_(__1__)_ = 417.62, *p* < 0.001, therefore justifying the treatment of the variables as separate constructs. However, it is noteworthy that the 2-factor model did not fulfill the criteria for acceptable model fit (cf. Schermelleh-Engel et al., 2003).

**TABLE 4:** Results of the confirmatory analyses testing the distinctions between emotional diabetes-related problems and symptoms of stress, respectively burnout.

		χ^2^	*df*	χ^2^/*df*	CFI	RMSEA	Δχ^2^	Δ*df*
Emotional DD and burnout	1-factor model	917.98	135	6.80	0.798	0.140	417.62***	1
	2-factor model	500.62	134	3.74	0.905	0.096		

***p < 0.001.

## Discussion

The present study’s findings on employees with type 1 diabetes show that DD is associated with burnout and job satisfaction. Higher levels of DD are linked to higher levels of burnout and lower level of job satisfaction. For each of the respective outcomes, a specific facet of DD explained variance in the outcome above and beyond job demands and control over time. For burnout, the emotional facet of DD (e.g., feeling alone with diabetes) was most relevant. In contrast, the social facet of DD (e.g., worrying about reactions) was strongly related to job satisfaction.

Additionally, it is important to emphasize the extent of the variance explained by diabetes-related variables found in this study. Previous research estimates the correlations between workplace-related stress and health variables to rarely exceed *r* = 0.333 or *R*^2^ = 0.10 (Faragher et al., 2005). In one of the two full models of our study (Model 1c), the DD explained more variance than age, gender, and workplace variables together, indicating that diabetes-related problems may have a greater impact on job satisfaction than job characteristics. The variance explanation of job satisfaction in Model 2c is smaller compared to the variance explanation of burnout (Model 1c). However, it is important to note that in Model 2c DD still explained 10% of the variance in job satisfaction. The insulin treatment method (pump vs. pen) did not affect burnout or job satisfaction, implying the rejection of Hypotheses 3a and 3b. However, pump users, in contrast to pen users, reported less food-related DD.

### Theoretical Implications

Our results align with findings on DD and burnout among people with diabetes type 2 (Han, 2008). This reinforces the assumption that health status should be viewed as an individual resource that plays an important role in the development of burnout (McGonagle et al., 2015). Previous research has been strongly focusing on physical health or illness as a critical outcome of burnout. Yet, systematic analyses on the exact causal nature of the association and, in particular, possible reciprocal effects are lacking (Maslach, 2001). There are, to our knowledge, no existing models of occupational health and burnout (e.g., JDR and COR) that account for a possible diversity in individual employee health statuses. Thus, our study is in line with previous work that suggests an integration of occupational health and diversity research, viewing chronic illness as a dimension of organizational diversity (Beatty and Joffe, 2006).

The associations found in this study support the proposition of investigating existing health status and health impairments as a personal resource within the JDR (McGonagle et al., 2015). Personal resources can directly impact well-being, moderate the effects of job characteristics, mediate the effect of job characteristics, and influence the perception of job characteristics (Schaufeli and Taris, 2014). Research on well-being fundamentally supports this general understanding of the impact of health (Sonnentag, 2015): The current level of well-being affects the perception of job demands (de Jonge et al., 2001), job resources (e.g., Reis et al., 2015) and personal resources (Xanthopoulou et al., 2009) in the future. Taking these findings into account, it can be assumed that health impairments like DD impact the well-being of chronic-ill employees and their perception of working conditions. This study mostly focused on the direct effect of diabetes-related problems (as a proxy for impaired health) on well-being outcomes. Future research should investigate whether individuals with diabetes-related problems or individuals with health impairments in general show different reactions to job characteristics in general terms of well-being outcomes.

Moreover, future research should analyze whether the subjective perception of job characteristics, such as quantitative and cognitive job demands, depends on the health status of the perceiver, for example by comparing employees with different health statuses working the same or very similar job within the same organization or team. Insights on these aspects allow a differentiate consideration of chronic-ill employees in their organizations. This is relevant in that illness management is predominantly the task of chronic-ill employees (Rak, 2014) and organizations should take more responsibility in this regard.

Further results of our study are that certain facets of DD are strongly related to burnout and job satisfaction. The emotional facet of DD (e.g., feeling alone with diabetes) was most relevant for burnout, whereas the social facet of DD (e.g., worrying about reactions) for job satisfaction. The strong association between the emotional facet of DD and burnout may be based on the common relationship to depression. Employees exposed to difficult working conditions for a long time have a higher risk for burnout, which can cause depression in the long term (Hakanen and Schaufeli, 2012). However, the measurement of emotional distress in diabetes and the psychiatric diagnosis of depression shows a conceptual overlap that requires a strong association (Gonzalez et al., 2011). The differences in the magnitude of explained variance between burnout and job satisfaction by the DD subdimensions is in line with the assumptions job satisfaction is a more distal outcome of work-related stressors, and a possible consequence of burnout (Wolpin et al., 1991). Thus, future studies with longitudinal designs should investigate the possibility of burnout mediating the effect of DD on job satisfaction.

Additionally, it can be assumed that employees in our sample have been confronted with the consequences of diabetes for a long time, which increases the emotional burden. The specific association between the social facet of DD and job satisfaction shows parallels to the relationship of neuroticism and job satisfaction, which is one of the strongest personality factors regarding job satisfaction (Judge et al., 2002). Employees with a high level of neuroticism are less satisfied with their work. The anxiety of reactions from other people is a crucial element of neuroticism and the social facet of DD. This might be an explanation for the specific association between the social facet of DD and job satisfaction.

In contrast to hypotheses 3a and b, the type of insulin therapy did not predict any work-related outcome. However, the portion of pen and pump users was relatively balanced in our sample. Despite the advantages of insulin pumps in the illness management of diabetes type 1 (Karges et al., 2017), employees did not experience less burnout or more job satisfaction than employees using an insulin pen. One possible reason for this result might be the habitation of handling with the respective insulin therapy over time. People get used to dealing with the type of insulin therapy and integrate them into their daily routine. An interesting finding in the additional analyses (Supplementary Appendix B) was that pen users reported more control over their work time than pump users. This may imply that the choice of insulin intake could be guided my aspects of the job itself. Less control over work time could imply more difficulties regarding diabetes management (i.e., monitoring blood glucose and manually injecting), thus providing a reason to choose a pump over a pen. Future research should consider this aspect when investigating the impact of insulin therapy among employees with diabetes type 1.

### Practical Implications

Our findings are highly relevant for employed or soon to be employed individuals with type 1 diabetes, diabetologists, and other diabetes-related treatment and counseling providers, as they are the main providers of diabetes-related information. Awareness of the strong associations between DD and burnout may prevent employees with high levels of burnout from using self-blame as a coping mechanism (Spataro et al., 2016) and motivate them to pay close attention to warning signs of exhaustion. Furthermore, knowledge about the effects of diabetes on work-life can be crucial for the career choices of adolescent people with type 1 diabetes.

Our results indicate that employees with type 1 diabetes have additional challenges that are associated with work-related outcomes. The reduction of DD provides an interesting starting point for improving work-life and preventing burnout among employees with diabetes type 1. Current approaches to working with diabetes mainly focus on the individual as the main actor in maintaining individual health and preventing worsening of the illness or it’s symptoms. A meta-analysis on self-efficacy education programs in persons with diabetes shows positive effects on HbA1C levels, self-management behaviors, knowledge, and quality of life. However, the review notes that most of the studies are characterized by low quality, short-term follow-up periods, and deficient physiological and emotional strategies (Jiang et al., 2019). The limited usage of strategies to improve the emotional state seems problematic as our findings show that the emotional facet of DD is particularly important for burnout. Other approaches, such as a specific 12-week coaching program for working individuals with chronic illness (e.g., ankylosing spondylitis, multiple sclerosis, nerve injury or neuropathy, diabetes Types 1 and 2), may provide a first starting point (McGonagle et al., 2014). This coaching intervention focuses on reinforcing four central personal resources in the context of work-related health (job self-efficacy, mental resources, core self-evaluations, and resilience) to reduce work-related challenges of employees with chronic illness (e.g., coming to work when sick, disclosing illness at work, long-term sickness absence and low levels of workplace support) and hence prevent further resource losses (Hobfoll, 1989).

In addition to the individual initiative of chronically ill employees to improve their illness management, organizations should support these efforts in workplace health management. However, organizations may be unaware of the prevalence of chronic illness among their employees, especially because symptoms are often invisible (Beatty and Joffe, 2006). In the context of diversity, organizations should explicitly name chronic ill employees as a significant part of the organizational workforce in the mission statement and point out that their specific concerns will be considered in organizational decision-making processes (e.g., design of tasks, workflow, and roles as well as health-related offers). Chronic illness can lead to day-to-day fluctuations of the employee’s capabilities. Thus, flexibility may be a core aspect in this process, for example, regarding work schedules (e.g., flextime), task assignments (e.g., completing tasks according to the present physical condition), and methods of task performance (e.g., work from home) (Beatty and Joffe, 2006). However, the respective actions must be planned and implemented with close regard to the needs and requirements of the chronically ill employees, which requires further investigation into the specific needs of employees with type 1 diabetes at work. A high level of organizational support may motivate chronically ill employees and evoke trust for illness disclosure leading to more inclusive organizations.

### Strengths and Limitations

A strength of the study lies in adequately sized sample of employees with a specific chronic illness which allows us to investigate associations between health- and work factors amongst people with type 1 diabetes. Chronic illnesses are still greatly overlooked in occupational health research and organizational diversity research (Beatty and Joffe, 2006). During the data collection period, we received positive feedback from participants that felt that the topic needs more attention, and several participants shared suggestions for future research topics that were rooted in their day-to-day experiences.

However, there are several limitations. First, the cross-sectional design does not allow to test for causal relations. Future studies should include prospective and longitudinal designs, e.g., to test for reciprocal effects of diabetes-related and work-related outcomes. Second, our study is based solely on self-reported data, which is why we cannot exclude the possibility of a common method bias. Further studies should combine self-reported questionnaire data with physiological indicators such as the HbA1c to increase the validity of the findings. As the inclusion criteria were also based on self-report, we cannot exclude the possibility that people without type 1 diabetes took part in the study. Although we announced the study both in social media and in a print outlet, the fact that we assessed the data via an online survey might have attracted a younger sample. Third, although the 4-factor solution of DD fit the data best, the overall fit of the model was not good and did not match the findings of previous studies on the factor structure of the PAID (e.g., Snoek et al., 2000). We also found less than acceptable fit indices regarding the 2-factor model of emotional DD and burnout. It is important to keep in mind that the PAID was initially developed as a unidimensional instrument, therefore further studies on the construct validity of the instrument should be carried out.

Finally, our burnout instrument assessed burnout mainly in terms of emotional exhaustion. Although emotional exhaustion is considered the core component of burnout as it is linked to physiological stress outcomes of the autonomic nervous system (Kanthak et al., 2017), as well as depression (Hakanen and Schaufeli, 2012), future research should investigate whether there are similar associations between DD and other subtypes of burnout. Furthermore, burnout and other measures of work-related well-being should be investigated as possible mediators for the association between type 1 diabetes and general well-being variables, such as depression.

## Conclusion

In sum, our analyses suggest that diabetes distress is meaningfully associated with burnout and job satisfaction among employed people with type 1 diabetes, thus providing one of the first pieces of evidence of a link between type 1 diabetes and negative work outcomes and supporting similar findings amongst people with type 2 diabetes. These findings can contribute theory and research on occupational health, diabetes counseling and treatment, and career and health coaching of people with type 1 diabetes.

## Data Availability Statement

The raw data supporting the conclusions of this article will be made available by the authors, without undue reservation, to any qualified researcher.

## Ethics Statement

Ethical review and approval was not required for the study on human participants in accordance with the local legislation and institutional requirements. The patients/participants provided their written informed consent to participate in this study.

## Author Contributions

AC co-wrote the theory section and hypotheses, designed the study, collected the data, analyzed the data, and wrote the first draft of the manuscript and guarantor of this work and, as such, had full access to all the data in the study and takes responsibility for the integrity of the data and the accuracy of the data analyses. AZ co-wrote the theory and discussion sections and reviewed and edited the manuscript. Both authors contributed to the article and approved the submitted version.

## Conflict of Interest

The authors declare that the research was conducted in the absence of any commercial or financial relationships that could be construed as a potential conflict of interest.

## Publisher’s Note

All claims expressed in this article are solely those of the authors and do not necessarily represent those of their affiliated organizations, or those of the publisher, the editors and the reviewers. Any product that may be evaluated in this article, or claim that may be made by its manufacturer, is not guaranteed or endorsed by the publisher.

## Footnotes

1 Due to the limited capacity of the local Ethics Review Board (ERB), researchers at faculty were asked to provide a description of the study design, as well as the participant information and the consent forms for a preliminary inspection. This pre-review concluded in the statement of the ERB that a full ethics proposal was not necessary.
