# Supplementary material for: Working With Type 1 Diabetes: Investigating the Associations Between Diabetes-Related Distress, Burnout, and Job Satisfaction
Source: Front Psychol. 2021 Nov 4;12:697833. doi: 10.3389/fpsyg.2021.697833 (PMC8599573; doi:10.3389/fpsyg.2021.697833)
Supplement: Supplementary file 1 [file Data_Sheet_1.docx]

Appendix A

*Results of two sample t-tests for differences between male and female participants*

|  | *M_Female_* | *M_Male_* | *df* | *t* |
| --- | --- | --- | --- | --- |
| Age | 38.96 | 45.87 | 295 | -5.26** |
| Quantitative job demands | 3.08 | 3.14 | 295 | -.56 |
| Control over work time | 3.26 | 3.75 | 295 | -3.71** |
| Emotional DD | 2.52 | 2.29 | 162.81^a^ | 1.92 |
| Social DD | 2.05 | 1.80 | 295 | 1.96 |
| Food-related DD | 2.53 | 2.30 | 295 | 1.87 |
| Therapy-related DD | 1.98 | 1.77 | 295 | 1.95 |
| Burnout | 3.15 | 2.78 | 295 | 3.58** |
| Job satisfaction | 3.47 | 3.57 | 295 | -1.05 |

*Note: N_female_* = 200, *N_male_* = 97, ** *p* > .01, ^a^Welch two sample t-test due to differences in variances between the groups

Appendix B

*Results of two sample t-tests for differences between male and female participants*

|  | *M_Pen_* | *M_Pump_* | *df* | *t* |
| --- | --- | --- | --- | --- |
| Age | 42.17 | 40.38 | 295 | 1.39 |
| Quantitative job demands | 3.12 | 3.08 | 295 | .47 |
| Control over work time | 3.56 | 3.30 | 295 | 2.01* |
| Emotional DD | 2.51 | 2.39 | 295 | 1.10 |
| Social DD | 1.95 | 1.98 | 295 | -.32 |
| Food-related DD | 2.66 | 2.83 | 295 | 3.27** |
| Therapy-related DD | 1.86 | 1.96 | 295 | -.92 |
| Burnout | 2.99 | 3.06 | 295 | -.81 |
| Job satisfaction | 3.54 | 3.48 | 295 | .65 |

*Note: N_Pen_* = 138, *N_Pump_* = 159, **p* < .05, ***p* < .01
